# Lumbar Annular High-Intensity Zone as a Precursor to Disc Extrusion

**DOI:** 10.7759/cureus.20111

**Published:** 2021-12-02

**Authors:** Deion L Ellis, Reza Ehsanian, Peter C Shin, William E Rivers

**Affiliations:** 1 Physical Medicine & Rehabilitation, University of New Mexico School of Medicine, Albuquerque, USA; 2 Neurosurgery, University of New Mexico School of Medicine, Albuquerque, USA; 3 Physical Medicine & Rehabilitation, Vanderbilt University Medical Center, Tennessee Valley Healthcare System, Department of Veterans Affairs, Nashville, USA

**Keywords:** annular fissure, high intensity zone, magnetic resonance imaging, intervertebral disc degeneration, intervertebral disc displacement, intervertebral disc disease, radiculopathy, herniated nucleus pulposus, low back pain

## Abstract

Low back pain (LBP) is a common affliction with numerous causes. Some individuals experience LBP attributed to disc pathology. Disc pathology has been implicated in a plurality of cases of LBP, and some cases are associated with annular fissures (AFs). AFs are weaknesses in the structure that contains the nucleus pulposus and is the site of possible disc herniations. On magnetic resonance imaging (MRI), some AFs manifest as the high-intensity zone (HIZ), otherwise known as an annular enhancement region. In this report, we present three patients with LBP, mild radiculitis, and HIZ who later developed herniated nucleus pulposus (HNP) with extrusion through the HIZ. These cases suggest that HIZ indicates a propensity for the future development of disc extrusion through the weakened tissue at the AF visualized as HIZ on MRI. With a better understanding of the association between AFs and disc herniations with HIZ, clinicians may be able to predict and prevent the pain and disability associated with disc extrusion.

## Introduction

Low back pain (LBP) is a common medical issue with a variety of underlying causes [1]. Disc pathology has been implicated in a plurality of cases of LBP [1], and some cases are associated with annular fissures (AFs). AFs are tears in the outer ligamentous ring of the intervertebral disc, called the annulus fibrosis. The annulus is composed of robust fibers, and the dorsal part of this structure is highly innervated with pain receptors [2-3]. In individuals with LBP secondary to disc pathology, discography is the gold standard for identifying which spinal disc is painful and whether AFs are present. However, on magnetic resonance imaging (MRI), some AFs manifest as the high-intensity zone (HIZ), otherwise known as an annular enhancement region [4-5].

An HIZ was first described in 1992 and was defined as a small, round zone with limited high-intensity signals in the posterior annulus of lumbar intervertebral discs on sagittal slices of T2-weighted MRI images [6]. A strong correlation between HIZ and positive provocative discography has been noted, indicating that HIZ is associated with discogenic back pain [6-8]. Histological analysis of HIZ revealed vascularized granulation tissue in the outer region of the annulus fibrosus, indicating a history of annular trauma and ongoing efforts of healing [9]. Inflammatory mediators found within AF have been associated with LBP and previous disc herniation [10-11]. While HIZ has been associated with LBP, it is also found less frequently in asymptomatic individuals [12-13]. There is a paucity of reports proposing lumbar annular HIZ as a precursor to disc extrusion. In this paper, we will present three patients with LBP, mild radiculitis, and HIZ without nerve compression who later developed disc extrusions through the HIZ causing nerve compression and symptoms of radiculitis.

## Case presentation

Case 1

**Figure 1:**
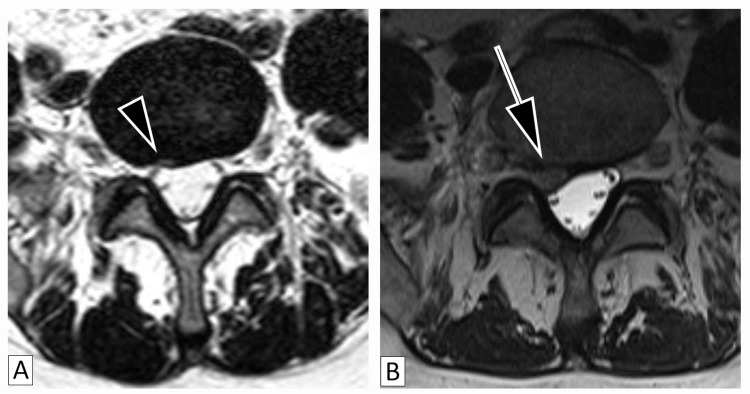
Magnetic Resonance Imaging of the High-Intensity Zone and Disc Extrusion Patient (Case 1) with magnetic resonance imaging revealing high-intensity zones (1A) indicated by the arrowhead correlating to areas of disc extrusion (1B) indicated by the arrow.

The first patient is a 64-year-old female with an over a decade-long history of chronic LBP lumbar spondylosis and facet arthropathy, which was successfully treated with multiple intraarticular steroid injections. In 2017, she presented to the clinic with acute exacerbation of LBP and was found to have an HIZ at the L5-S1 disc (Figure 1A), which was treated with physical therapy and multiple intraarticular steroid injections. In 2019, she was referred to our clinic due to exacerbation of LBP with radiation of pain into the right buttock without other neurological signs or symptoms. MRI demonstrated right L5-S1 subarticular disc extrusion from the location of the HIZ with compression of the right traversing S1 nerve root (Figure 1B). Her radicular symptoms in the back and buttock remitted after several months of physical therapy and anti-inflammatories.

Case 2

**Figure 2:**
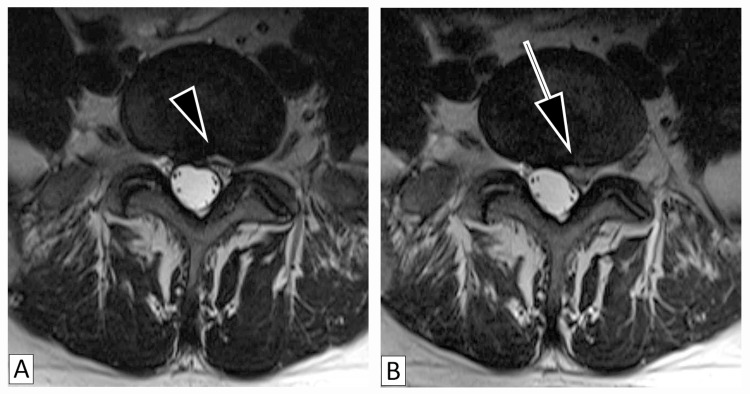
Magnetic Resonance Imaging of the High-Intensity Zone and Disc Extrusion Patient (Case 2) with magnetic resonance imaging revealing high-intensity zones (2A) indicated by the small arrowhead correlating to areas of disc extrusion (2B) indicated by the arrow.

The second patient is a 53-year-old male with an over a decade-long history of chronic LBP and left radicular pain, which was treated conservatively with physical therapy and epidural steroid injection with partial remission of symptoms. In 2018, he presented to the clinic with acute exacerbation of LBP and was found to have an HIZ in proximity to the left S1 nerve root at the L5-S1 disc (Figure 2A), which was treated with physical therapy and epidural steroid injections. In 2020, he returned to the clinic with increased LBP and left radicular pain radiating into the plantar aspect of the foot without neurological signs. MRI demonstrated disc extrusion at L5-S1 from the location of the HIZ impinging on the left traversing S1 nerve root (Figure 2B). His radicular symptoms remitted after several months of physical therapy, anti-inflammatories, and epidural steroid injections.

Case 3

**Figure 3:**
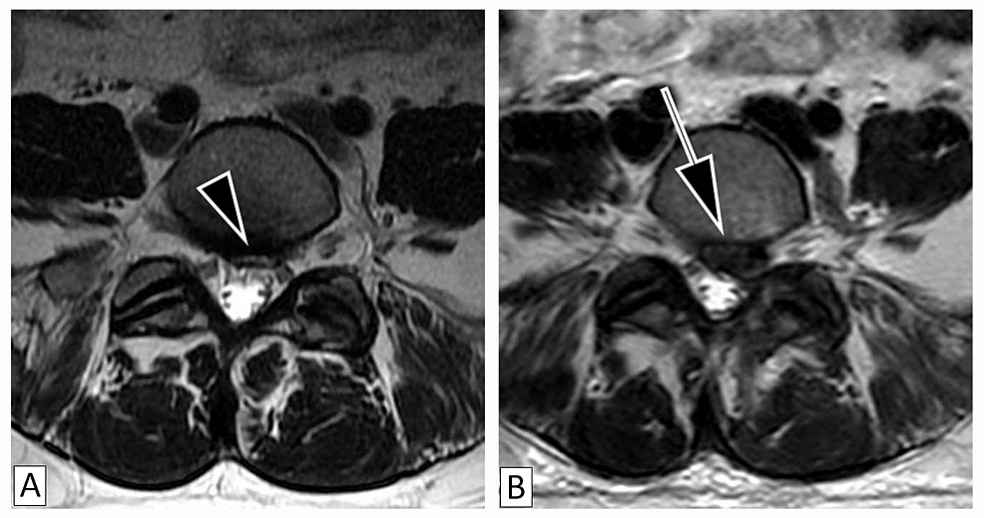
Magnetic Resonance Imaging of the High-Intensity Zone and Disc Extrusion Patient (Case 3) with magnetic resonance imaging revealing high-intensity zones (2A) indicated by the small arrowhead correlating to areas of disc extrusion (2B) indicated by the arrow.

The third patient is a 51-year-old female diagnosed with systemic lupus erythematosus and a five-year history of chronic LBP associated with left radicular leg pain radiating to the first digit of her foot, which was managed conservatively with physical therapy, epidural steroid injections, muscle relaxants, nonsteroidal anti-inflammatories, and opioids. In 2018, she presented due to minimal improvement of her LBP and was found to have an HIZ at the L5-S1 disc without any compression of nerve roots (Figure 3A), which was treated conservatively. In 2019, she had worsening radicular symptoms without other neurological signs. MRI demonstrated disc extrusion at L5-S1 from the location of the HIZ (Figure 3B). Her symptoms did not respond to physical therapy, medication management, including increased doses of her medications, or epidural steroid injections, so she underwent surgical discectomy for decompression.

## Discussion

LBP caused by disc pain is a common medical issue [1]. MRI is commonly used in the diagnosis of patients with LBP [5]. HIZ is one of the MRI findings that may reflect a painful structure. However, the association between HIZs and LBP continues to be under debate [2-3]. This is often due to discrepancies between the clinical profile and MRI findings [14].

The finding of HIZ on MRI is reflective of AF [6-7]. However, not all AFs have corresponding HIZ on MRI, and not all HIZ reflect AF. CT discography remains the standard for identifying AF since the injection of contrast with subsequent cross-sectional imaging can verify the presence of AF with leakage of contrast into the anatomical defect [12,14]; these defects are not always visible as HIZ on MRI. It has been previously suggested that AF plays a role in LBP due to inflammation and neo-neurogenesis within granulation tissue [10].

AF pain may respond to surgical debridement of inflammatory granulation tissue, ablation of neural ingrowth, and removal of local friable nucleus pulposus fragments [15-17]. Supporting this contention, specific histological and discoscopic findings are reported in patients with HIZ that correspond to specific degenerative changes of vascular ingrowth, cartilaginous insufficiency, and fibroblast proliferation, which may result in pain and dysfunction [7,16]. Accordingly, two large case series of an endoscopic laser-assisted discectomy with annuloplasty to address chronic LBP with AF (identified using fluoroscopic guided discogram but not by HIZ) showed a substantial reduction in average pain score and back-pain associated disability [16-17]. An additional group retrospectively reviewed cases from among many patients with diverse lumbar spinal pathologies who received a similar treatment and noted 43.3% reported “good” or “excellent” outcomes [15]. These favorable responses to a rational anatomical treatment support the link between AF and LBP.

In addition to their role in LBP, AFs are weaknesses in the circumferential annulus fibrosus structure that contains the nucleus pulposus [4-5]. It should be noted that all disc herniations occur through AF, with the exception of Schmorl’s nodes where the nucleus pulposus violates the cortex of the vertebral body. HIZ commonly indicates a current AF or residual tissue after a previous herniated nucleus pulposus (HNP) has started to heal [9]. Notably, in the case series from Ahn and Lee, the presence of HNP with AF was associated with a higher rate of success of treatment, further reinforcing the interdependence of AF, HNP, and LBP [17].

Is there a role for the surgical debridement of AF and underlying nucleus pulposus for chronic and debilitating LBP, with the intention of preventing future HNP with radiculopathy? Debriding the annular and nuclear material associated with the AF may treat the debilitating axial LBP condition and, furthermore, may prevent the recognized debility associated with radicular leg pain from compression of the nerve root caused by HNP. On the other hand, there are three major reasons not to rush to operation to prevent HNP at the site of AF. First, not all AF with LBP progress to symptomatic HNP with radiculopathy. Second, radiculopathic symptoms commonly remit without operative intervention, especially from extruded HNP, as demonstrated by accumulated data on the natural history of the condition and further illustrated by the patients in Case 1 and Case 2 above, whose HNP-related radicular symptoms remitted after several months of conservative therapy [18]. Third, operative risk includes nerve trauma, increased LBP, and disc herniation through the operative defect, all of which may cause further pain and disability for the patient [19]. As always, careful clinical consideration of the risks and potential benefits of possible treatments is required, with full and informed consent from the patient.

## Conclusions

Since the original description of HIZs in the 1990s, considerable interest has surrounded this imaging finding and its utility. In patients with LBP, HIZ predicts a positive discogram result. Several groups have demonstrated that a subset of patients with LBP and AF respond to annuloplasty and discectomy with significant remission of pain and disability, further strengthening the clinical utility of the correlation and offering a possible treatment for the severe low back pain associated with this condition.

The cases presented in this report suggest that HIZ indicates a propensity for the future development of disc extrusion through the weakened tissue at the location of the AF represented by the HIZ. Understanding this association should lead the clinician to suspect disc extrusion among patients with known HIZ whose symptoms suddenly change in character, intensity, or distribution. Further research is warranted to better understand the association between AF, HNP, and HIZ, in the hopes of predicting, preventing, and treating the pain and disability associated with AF and disc extrusion.
